# Clinically significant avoidance of public transport following the London bombings: Travel phobia or subthreshold posttraumatic stress disorder?

**DOI:** 10.1016/j.janxdis.2009.07.023

**Published:** 2009-12

**Authors:** Rachel V. Handley, Paul M. Salkovskis, Peter Scragg, Anke Ehlers

**Affiliations:** a King's College London, Institute of Psychiatry, Department of Psychology, and Centre for Anxiety Disorders and Trauma, London, UK; b Trauma Clinic, London, UK

**Keywords:** Terrorist violence, Specific phobia, Posttraumatic stress disorder, Screening

## Abstract

Following the London bombings of 7 July 2005 a “screen and treat” program was set up with the aim of providing rapid treatment for psychological responses in individuals directly affected. The present study found that 45% of the 596 respondents to the screening program reported phobic fear of public transport in a screening questionnaire. The screening program identified 255 bombing survivors who needed treatment for a psychological disorder. Of these, 20 (8%) suffered from clinically significant travel phobia. However, many of these individuals also reported symptoms of posttraumatic stress disorder [PTSD]. Comparisons between the travel phobia group and a sex-matched group of bombing survivors with PTSD showed that the travel phobic group reported fewer re-experiencing and arousal symptoms on the Trauma Screening Questionnaire (Brewin et al., 2002). The only PTSD symptoms that differentiated the groups were anger problems and feeling upset by reminders of the bombings. There was no difference between the groups in the reported severity of trauma or in presence of daily transport difficulties. Implications of these results for future trauma response are discussed.

## 1 Introduction

Large-scale traumatic events, such as natural disasters and terrorist attacks, are a challenge for mental health services. Previous studies have found that there are high rates of mental disorders amongst direct survivors of terrorist attacks (Whalley & Brewin, 2007). The response to such events involves identifying those in need of immediate help (and those who may need it later) and deploying appropriate support and expertise. This response will depend in large part on our understanding of the way in which the traumatic event is likely to have affected those caught up in it. At the most basic level, this means screening for clinically significant psychological problems so that appropriate treatment can be initiated. Most research has concentrated on screening for posttraumatic stress disorder (PTSD; Brewin, 2005). There is, however, increasing awareness that other disorders such as depression and specific phobias are also common consequences of trauma (for a review see Brady, Killeen, Brewerton, & Lucerini, 2000).

The present study focuses on the identification of travel phobia following the terrorist attacks on public transport in London. On 7 July 2005, four terrorist bombs exploded on the London transportation system. Three were detonated on underground trains at three different stations and one on a bus at a square in Central London. Fifty-two people were killed in the attacks and more than 775 injured from among the more than 4000 passengers involved.

Surveys of the general population in London in the aftermath of the bombings (e.g., Rubin et al., 2007) indicated some persistent low-intensity changes in travel behaviour following the bombings. Persistent changes in travel behavior were identified as any reduction in travel by tube, train, bus and car as a result of the bombings that were reported both in July 2005 and at follow-up in February/March 2006. We expected that a proportion of those directly involved in the bombings would develop clinically significant travel phobia.

### 1.1 Travel phobia and PTSD after trauma

The key symptoms of travel phobia are excessive fear and avoidance of travel situations. These symptoms overlap with those of PTSD. In particular, persistent avoidance of stimuli associated with the trauma and fear and other negative emotions in response to trauma reminders are common PTSD symptoms. This raises the question of how well travel phobia after experiencing a traumatic event can be distinguished from PTSD.

It is possible that travel phobia following trauma is just a (milder) version of PTSD. The *Diagnostic and Statistical Manual of Mental Disorders* (DSM-IV, American Psychiatric Association, 1994) takes this view. DSM-IV stipulates that the diagnosis of specific phobia should not be given if the phobic anxiety and avoidance is better accounted for by PTSD. In practice this means when fear and avoidance *only* occur in trauma related situations, and the other PTSD criteria are met, a diagnosis of PTSD rather than specific phobia is considered appropriate. Furthermore, there is evidence that trauma survivors with subthreshold PTSD, i.e. those who meet most, but not all DSM-IV criteria for PTSD, may suffer clinically significant impairment (e.g., Stein, Walker, Hazen, & Forde, 1997; Weiss et al., 1992; Zlotnick, Franklin, & Zimmerman, 2002). This raises the question of whether phobic reactions after trauma may represent a form of subthreshold PTSD.

However, there is also evidence that phobias and PTSD may constitute correlated but distinct responses to trauma. Mayou, Bryant, & Ehlers (2001), for example, conducted a prospective study of psychological outcomes after motor vehicle accidents. While they found that PTSD and phobic travel anxiety (as well as general anxiety and depression) often overlapped they could also occur separately and had different onset and course. Predictor variables also overlapped but there were some differences. For example, phobic responses, but not PTSD, were predicted by mode of transportation, with passengers having a greater risk of subsequent travel phobia than drivers. In contrast, cognitive and social factors measured 3 months after the accident such as rumination and negative interpretations were strong predictors of PTSD. Kleim, Ehlers, and Glucksman (submitted for publication) used structural equation modeling to examine the latent structure of symptoms of PTSD, specific phobia, and depression in a sample of assault survivors and found that a model of three correlated but distinct outcomes fitted the data better than a unitary model of post-trauma psychopathology.

Thus, it is at present unclear whether travel phobia can develop after traumatic events and whether it can be distinguished from PTSD. The purpose of the present study is to examine these issues in survivors of the London bombings of 7 July 2005 (7/7).

### 1.2 Mental health services response to the London bombings

Following the bombings in July 2005, a Screening Team was established in September (under the guidance of a Psychosocial Steering Group), to screen individuals directly involved in the London bombings for a variety of psychological problems commonly observed following trauma, including travel phobia and PTSD, and to offer prompt referrals for treatment. Existing trauma services across London increased their capacity so that those identified by the Screening Team could be treated without delay. The program and initial treatment outcome are described more fully elsewhere (Brewin, Scragg, Robertson, Thompson, & Ehlers, 2008). The present paper focuses on those identified by the Screening Team as suffering from clinically significant travel phobia.

### 1.3 Aims of the study

The first aim of the study was to identify the proportion of people directly involved in the London bombings (a) who reported clinically significant specific phobia of public transport in a screening questionnaire and (b) who needed treatment for travel phobia rather than for other post-trauma reactions. The second aim was to investigate what distinguishes individuals presenting with travel phobia from those presenting with PTSD: in particular, whether the differences could be picked up by a self-report screening questionnaire. As intrusive memories have been described as the “hallmark” symptom (Foa, Steketee, & Rothbaum, 1989), or the “core” of PTSD (Steil & Ehlers, 2000) it was hypothesized that trauma survivors with travel phobia would be less likely to endorse re-experiencing symptoms than those with PTSD. It was also hypothesized that the PTSD group would report a greater severity of their traumatic experience.

## 2 Method

### 2.1 Overview

In an outreach program, people directly involved in the London bombings of 7 July 2005 were contacted and asked to fill in a questionnaire screening for PTSD, travel phobia and other symptoms. Those scoring positive on the screener were invited for a brief structured diagnostic interview. The present paper reports on the individuals identified at this brief interview as suffering from clinically significant travel phobia in response to the bombings. These were compared with a sex-matched comparison group identified as suffering from PTSD by the same screening process.

### 2.2 Outreach program

The Screening Team received referrals from a variety of organizations and individuals that had had contact with survivors of 7/7 including Accident and Emergency Departments, the Metropolitan Police Witness List, the Health Protection Agency, the Family Assistance Centre, 7th July Assistance Centre, NHS direct helpline, and family doctors. In addition, some individuals or their friends and relatives contacted the Screening Team directly when they saw the details advertised in the media.

### 2.3 Screening questionnaire

The Screening Team sent the bombing survivors a two-page questionnaire comprising four sections. The first section established demographic information. The second section comprised questions designed to measure the severity of the participant's traumatic experience. Subjective severity was defined as the sum of the following two items (scored as ‘0’ absent and ‘1’ present): the person thought they might be injured or killed; they felt that a family member or close friend might be injured or killed. Objective severity was defined as the sum of the following five items (scored as ‘0’ absent and ‘1’ present): the person was injured; saw someone who had been injured or killed; a family member or close friend was injured; a family member or close friend was killed; they personally witnessed the effects of one of the bombings.

The third section comprised the Trauma Screening Questionnaire (TSQ; Brewin et al., 2002). The TSQ is a tick list screener for the symptoms of the re-experiencing and arousal identified by criterion B and D of the DSM-IV (American Psychiatric Association, 1994) PTSD diagnosis, i.e., intrusive, distressing thoughts or memories, upsetting dreams, flashbacks, upset at reminders, bodily reactions with reminders, sleep difficulties, anger, concentration problems, hypervigilance and startle reactions. Participants were asked to endorse those symptoms that they had experienced at least twice in the past week.

The fourth section comprised screening questions for other disorders. The travel phobia screener question was as follows: “Since the bombings, has your daily life become difficult because you felt unable to use public transport (e.g. not being able to get to work, to get your shopping done or get to social events), or because you felt very distressed when using public transport?” Other screener questions related to depression (low mood and loss of interest), substance abuse (increased tobacco or alcohol consumption) and “other” unspecified reactions.

### 2.4 Diagnostic interviews and treatment referral

Respondents were invited for diagnostic interview if they either endorsed (i) six or more items of the TSQ, (ii) the travel phobia item or (iii) the two depression items on the screening questionnaire. The diagnostic interview comprised the relevant modules (PTSD, specific phobia and depression) from the Structured Clinical Interview for DSM-IV (SCID; First, Spitzer, Gibbon, & Williams, 1997), and additional questions to assess alcohol use, pain and grief. The majority of individuals identified by the Screening Team as needing treatment for travel phobia was to be referred to and treated at the Centre for Anxiety Disorders and Trauma (CADAT), London. However, in some cases this was not possible; due to the very nature of their problems, some individuals were unable to travel to the CADAT and received treatment at services nearer to where they lived or worked.

Before starting treatment at the CADAT, participants were re-interviewed by their therapist, using the full SCID (First et al., 1997) and the Clinician Administered PTSD Scale (CAPS; Blake et al., 1990). Using these detailed assessment interviews, it was possible to determine to what extent the patients’ clinical presentation met a range of criteria for PTSD, including DSM-IV criteria, ICD-10 research criteria (re-experiencing, avoidance and either memory gaps or arousal symptoms; World Health Organization, 1993) or ICD-10 diagnostic criteria where re-experiencing is the only necessary symptom but a variety of other PTSD symptoms are typically present; World Health Organization, 1992).

### 2.5 Participants

Participants were drawn from the 596 respondents to the London bombing screen and treat program. Of the 346 survivors who attended the screening interview; 20 were diagnosed as suffering from a clinically significant travel phobia. The comparison group comprised 20 patients who were selected from those diagnosed with clinically significant PTSD at the screening interview, by recruiting the next survivor with PTSD of the same sex whenever a survivor with travel phobia was identified.

### 2.6 Data analysis

Group comparisons between the Travel Phobia and the sex-matched PTSD group were made using *t*-tests for continuous data and Chi Square or Fisher's exact test (as appropriate) for categorical data. Significance level was set at *α* = .05, two-tailed.

## 3 Results

### 3.1 Prevalence of travel anxiety and phobia

#### 3.1.1 Screening questionnaire

Five hundred and ninety-six initial screening questionnaires were returned to the Screening Team between July 2005 and May 2007 (Brewin et al., 2008). Forty-five percent of those returning a screening questionnaire endorsed the travel phobia item. Of the 314 people who had both attended a detailed assessment with the Screening Team and had completed a screening questionnaire (an additional 32 had been referred to the service by other means) 69% had endorsed the travel phobia screening item. All of those meeting criteria for Travel Phobia at screening assessment (20/20) and 72% of those meeting criteria for PTSD (120/166 with screening questionnaires) had endorsed the travel phobia item on their screening questionnaires. This difference was significant (*χ*^2^ = 5.96, *p* = .015). Notably, 27% of the people who endorsed the travel phobia item on the screening questionnaire (70/260) did not respond affirmatively to invitations to assessment interview. While 18 of these reported that they were receiving treatment elsewhere and a few were impossible to contact (4), the majority either did not respond at all (28), did not attend or cancelled appointments (11) or declined appointments altogether (9).

#### 3.1.2 Screening assessment

Three hundred and seventy individuals were invited for interview of whom 24 (6%) did not attend. Of the 346 individuals attending an assessment, 255 (74%) were found to require treatment. Twenty individuals (8%) met criteria for Travel Phobia at interview, most of whom were women (19/20, 95%). The most common diagnosis was a DSM-IV (American Psychiatric Association, 1994) or ICD-10 (World Health Organization, 1992) diagnosis of PTSD, *n* = 184 (72%).

### 3.2 Differences between Travel Phobia and PTSD groups

Participants presenting with Travel Phobia did not differ from the sex-matched PTSD group in terms of age (see Table 1), whether they had children or not (30% vs. 25%, n.s.) and site of the trauma.

Tables 1 and 2 show comparisons of the Travel Phobia and PTSD groups on the screening questionnaire. There were no differences between the groups in objective and subjective trauma severity (Table 1). There were also no group differences in the percentage of participants reporting daily difficulties when traveling (Table 2). The main difference between the groups was that the PTSD group reported more PTSD symptoms overall, which was due to both more re-experiencing symptoms and more hyper-arousal symptoms (Table 1). However, the travel phobia group was not less likely than the PTSD group to meet the DSM-IV criteria of least one re-experiencing symptom or two hyper-arousal symptoms (Table 2) according the screening questionnaire. There was a significant group difference in the proportion of participants who met the recommended screening questionnaire criterion of at least six symptoms (Brewin et al., 2002; Table 2) with fewer individuals in the travel phobia group meeting the screening criterion. However, a majority of 13 (65%) of the travel phobia group screened positive on the TSQ.

Two items of the TSQ distinguished between the Travel Phobia and PTSD groups. The Travel Phobia group was less likely to report anger and being upset by reminders (Table 2).

### 3.3 Further information obtained during treatment

Twelve of the Travel Phobia group were referred to CADAT and 11 attended the further SCID and CAPS assessments with their therapist and treatment. The majority reported some PTSD symptoms in the CAPS. According to the CAPS, two patients met DSM-IV criteria for PTSD at the clinical assessment. Three patients met ICD-10 research criteria for PTSD and four met ICD-10 diagnostic criteria for PTSD. Only two patients did not describe significant PTSD symptoms.

Treatment was adjusted to take the presence of PTSD symptoms into account. The treatment protocol and responses are described in greater detail in another paper (Handley, Salkovskis, & Ehlers, 2009). Of those travel phobia patients endorsing less than six items on the TSQ and subsequently referred to the CADAT (four patients), two (50%) required the PTSD treatment.

## 4 Discussion

### 4.1 Summary of results

The first aim of the study was to identify the proportion of people directly involved in the London bombings (a) who reported clinically significant specific phobia of public transport in a screening questionnaire and (b) who needed treatment for travel phobia rather than for other post-trauma reactions. An outreach screen and treat program was set up after the bombings. Of the 596 respondents to the screening program, 45% reported phobic fear of public transport in a screening questionnaire. The program identified 255 survivors who needed treatment, and 8% of these had a main diagnosis of travel phobia. As hypothesized this travel phobic group reported fewer re-experiencing and arousal symptoms and less PTSD symptoms overall compared to those referred for treatment of PTSD. The only individual symptom that distinguished the groups was that the travel phobic group was less likely than the PTSD group to report anger problems or feeling upset by reminders of the bombings. However, contrary to hypothesis the travel phobic group did not experience the trauma as less severe. There was no difference between groups in the presence of daily transport difficulties.

### 4.2 Travel anxiety and phobia among bombing survivors

The results of the screening questionnaire showed that travel anxiety was a common problem among those who responded to the screen and treat outreach program. Forty-five percent reported disabling travel anxiety that had interfered with their everyday life. These results are consistent with surveys of the general population in London suggesting wide spread fears and avoidance of travel in the aftermath of the bombing (Rubin et al., 2007).

However, in only a minority of the 8% of the participants who were identified as needing treatment at screening interview, travel phobia was the main problem. It was common for the travel fears to be part of a wider range of symptoms of PTSD or depression. The PTSD group reported being as disabled in their everyday life by fear and avoidance of public transport as the travel phobia group.

The relatively low prevalence of travel phobia among those needing treatment raised the question of whether the study may have underestimated the true prevalence of travel phobia. It is possible that some bombing survivors with travel phobia may not have contacted the Screening Team for a range of reasons, for example because of extensive avoidance or because they did not wish to seek treatment. In our experience, it was not uncommon for the survivors of the bombings to downplay their difficulties initially in treatment and to state that they felt undeserving of treatment compared with individuals who had, for example, been seriously physically injured or lost a limb as a result of the bombings. Furthermore, 48 individuals endorsing the travel phobia item in the screening questionnaire either declined or did not respond to the offer of assessment and 18 received treatment or support elsewhere.

Alternatively, the low prevalence of travel phobia among bombing survivors needing treatment may reflect a true pattern. A previous study of the development of phobia in Germans as a result of the September 11 bombings (Muhlberger, Alpers, & Pauli, 2005) suggested that despite a decrease in numbers flying there was no increase in fear of flying. There may be a qualitative difference in normative population behavior seen as a “sensible” avoidance of increased risk at times of state sanctioned increased security and media warnings, etc. and avoidant behavior that is driven by pathological fear.

The relatively low prevalence of clinically significant travel phobia is also consistent with the literature on the onset of phobias (Davey, 1995) which shows that the typical age of onset of phobias is pre-pubertal. Most passengers on the affected tubes and bus were adults and may have therefore been less vulnerable to developing phobic responses.

### 4.3 Screening for Travel Phobia and PTSD

The screening questionnaire used in this study was able to distinguish between those identified as having clinically significant travel phobia and those with PTSD, in that, consistent with diagnostic criteria, the travel phobia group endorsed fewer PTSD symptoms. However, differences were less clear cut than one may have expected. The majority of travel phobia cases screened positive on the TSQ and reported having six or more re-experiencing or arousal symptoms at least twice per week.

Although the PTSD group endorsed more re-experiencing symptoms on the screening questionnaire (which are widely considered the most characteristic symptoms of PTSD), the travel phobia group also endorsed re-experiencing items, but to a lesser extent. This result is consistent with recent literature suggesting that intrusive imagery involving phobic stimuli, often linked with distressing memories, is common in many types of phobias including, for example, snake phobia (Hunt et al., 2006), social phobia (Hackmann, Clark, & McManus, 2000) and agoraphobia (Day, Holmes, & Hackmann, 2004). Overall, these findings are consistent with the emerging view that intrusive imagery of past traumatic or feared events is not confined to PTSD.

There were only two items on the screening questionnaire that were specific to the PTSD group and were rarely endorsed by the travel phobia group, namely anger and being upset by reminders of the bombings. The difference in anger symptoms may point to differences in underlying cognitions and cognitive processes. Anger may be indicative of broader negative underlying cognitions and beliefs in the PTSD group around the trauma and their subsequent symptoms. Persistent anger may be underpinned by cognitions such as “what happened to me was not fair” or “the perpetrators of this event will never be punished whereas I am still suffering.” In contrast, the cognitions of the travel phobia group may be confined to negative beliefs about the threat of transport. Similarly, Ehlers, Mayou, and Bryant (1998) found that anger cognitions predicted PTSD in motor vehicle accident survivors, and Ehring, Ehlers, and Glucksman (2006, 2008) reported negative cognitions regarding the danger of travel and future accidents predicted travel phobia after accidents.

The group difference in being upset about reminders may be related to differences in avoidance patterns. It is possible that the travel phobia group may have been *less* avoidant of general reminders of the bombings (such as newspaper articles, TV programs or conversations). It is also possible that the travel phobia group was avoiding transport *more* than the PTSD group and therefore encountering fewer reminders of the event to become upset about.

Results of the screener questionnaire did not support the view that stressor severity is an important determinant in whether people develop a phobic response or PTSD in response to a traumatic event. There were no differences between the groups in objective and subjective trauma severity. However, group differences may have been blurred by the fact that the travel phobia group also reported many PTSD symptoms. Furthermore, the “subjective” severity items used in the questionnaire relate neither to negative appraisals after the trauma relating to the event itself nor to the symptoms experienced in its aftermath. There is evidence that such negative appraisals of the trauma and its sequelae are good predictors of subsequent PTSD (Dunmore, Clark, & Ehlers, 2001; Ehlers et al., 1998; Ehring et al., 2006, 2008; Kleim, Ehlers, & Glucksman, 2007).

### 4.4 Diagnostic Issues

When patients in the travel phobia group were re-assessed by their therapist prior to treatment, all except two also reported PTSD symptoms severe enough to meet at least ICD-10 diagnostic criteria for PTSD (Handley et al., 2009). This raises diagnostic and clinical issues. First, at a diagnostic level, it may be best to conceptualize these patients as suffering from partial PTSD (e.g., Stein et al., 1997; Weiss et al., 1992; Zlotnick et al., 2002) rather than a specific phobia. It also suggests that simple exposure to travel situations would not necessarily be the most appropriate intervention for the majority of cases. Current evidence suggests that trauma-focused CBT is the recommended treatment for PTSD (NICE, 2005). Individual case formulations that take into account partial PTSD symptoms and related cognitive processes are therefore needed (Handley et al., 2009).

It appeared that patients reported somewhat more PTSD symptoms when re-interviewed by their therapist than in the briefer screening interview. There may be several reasons. It is possible that the patient’ substantial avoidance initially led to an underestimation of PTSD symptoms as these patients may have successfully avoided important triggers of re-experiencing and arousal symptoms, and that the avoidance may have decreased with the commitment to therapy. It is also possible that patients were initially reluctant to endorse some of the PTSD symptoms and only felt ready to report them in treatment when they had had time to build up trust with the interviewer rather than in the shorter screening interview.

### 4.5 Limitations and future research

The major limitation to this study concerns the sampling of the population. Although efforts were made to contact everyone who was directly involved in the London bombings, only those who were interested in further screening and treatment could be assessed. It is not known if there are individuals who have been experiencing travel anxieties who did not wish to present for treatment. Future research could directly target these individuals for a more detailed assessment. Furthermore, not all individuals identified as needing treatment for travel phobia were referred to our clinic and thus only a subgroup was re-interviewed with the CAPS. However, given the nature of the problem studied and the massive effort to set up an unprecedented service for a large-scale trauma response in this country it was difficult to see the patients at one site. This study was complementary to this larger treatment effort and was designed alongside it with the primary aim of identifying and responding to those requiring treatment for travel phobia. It was, however, beyond available resources to identify and follow-up those who did not wish to have treatment.

For practical reasons, the screening questionnaire used in the screen and treat program could only contain one item relating to travel fears. Thus, it was not possible to distinguish on the screener between very pervasive travel phobias such as inability to travel alone, and milder forms where travel was still possible. More detailed items distinguishing travel fears, travel avoidance and interference and distress caused by travel could be included in further screening studies.

The study was exploratory, and only a relatively small group of patients needing treatment for travel phobia were identified. Due to low power, we could not correct for multiple testing and the results presented in the paper may therefore somewhat overestimate the ability of the screening questionnaire to distinguish between PTSD and travel phobia.

Finally, a further comparison with a group of survivors who did not suffer from fear and avoidance of transport following their experience of the bombings would have been an interesting extension to the present study. This group may have displayed other symptoms of PTSD and, if so, provided more insight into the pathways that determine which symptoms are most likely to occur for which individuals. Thus a comparison of a representative sample of those reporting avoidance of transport and those not reporting avoidance could have been interviewed, clustered according to the other symptoms displayed and these groups compared on variables such as severity of trauma and cognitive predictors of PTSD. Once again, given the speed, size and nature of the response it was not possible to attempt this methodology and this was largely an exploratory study.

### 4.6 Conclusions

A screening program that focused on those who were directly affected by the London bombings found that among those survivors who needed treatment, 8% presented with clinically significant travel phobia. However, close examination of the symptoms reported suggests that the majority also had additional PTSD symptoms. A travel phobic presentation after trauma while traveling is characterized by fear and avoidance of transport situations but individuals may also report intrusive memories of traumatic onset events and hyper-arousal symptoms. Thus, treatment of these phobic responses needs to include some elements of the treatment of PTSD to successfully treat the re-experiencing symptoms (Handley et al., 2009; Ehlers & Clark, 2000).

Sadly there have been continuing reports of terrorist plots and attempts to bomb trains and planes across the globe. Indeed, in some countries there is a constant, frequently realized threat of suicide bombers on buses and public transport. If future attacks of this nature occur, the results of the present study suggest that clinicians responding to the needs of survivors of these events should be aware of the possibility of clinically significant phobic responses both in the context of PTSD and as a separate problem. The use of valid and reliable screening instruments is important for the identification of those likely to be in need of treatment for these problems. Further work on developing sensitive and specific screeners for phobic responses after trauma is needed.

Subsequent thorough clinical interviews should pay attention to the discrimination between diagnoses, as these are important to guide service provision and clinical interventions. General population research and further surveys of individuals more closely involved with the events but not presenting for treatment should be conducted to identify the true magnitude of phobic responses. These problems may be exerting a significant influence on public health and well-being and it may be that individuals who do not wish to present to mental health services for treatment could be provided with help in other forums such as public workshops addressing travel anxieties.

## Figures and Tables

**Table 1:** Comparisons between the Travel Phobia and PTSD groups on demographic characteristics, symptom clusters, and symptom severity in screener questionnaire (means, standard deviations and *t*-tests).

	Travel phobia^a^		PTSD^a^		*t*-Test^b^	*p*
	*M*	SD	*M*	SD		
Age	36.50	11.80	36.75	10.39	.0710	.944
Total number of PTSD symptoms	6.30	2.18	8.35	1.31	3.607	.001**
Total number of re-experiencing symptoms	3.20	1.64	4.10	.91	2.143	.039*
Total number of arousal symptoms	3.10	1.21	4.25	.91	3.397	.002**
Subjective severity of trauma	1.05	.61	1.20	.62	−.777	.442
Objective severity of trauma	2.05	.83	2.25	.72	−.818	.418

a *n* = 20 for each group.

b d.f. = 38.

* *p* < .05.

** *p* < .01.

**Table 2:** Group differences in individual items of screener questionnaire and endorsing six or more items in total.

		Travel phobia^a^		PTSD^a^		Fisher's exact test
		*n*	%	*n*	%	
Daily transport difficulties	Absent	2	10	4	20	.661
	Present	18	90	16	80	

Meets cutoff of six symptoms on TSQ^b^	Absent	7	35	0	0	.008**
	Present	13	65	20	100	

Intrusive thoughts	Absent	5	25	1	5	.182
	Present	15	75	19	95	

Upsetting dreams	Absent	11	55	10	50	1.00
	Present	9	45	10	50	

Feels happening again	Absent	9	45	5	25	.320
	Present	11	55	15	75	

Upset by reminders	Absent	5	25	0	0	.047*
	Present	15	75	20	100	

Bodily reactions	Absent	6	30	2	10	.235
	Present	14	70	18	90	

Any re-experiencing symptom	Absent	2	10	0	0	.487
	Present	18	90	20	100	

Sleep difficulties	Absent	9	45	3	15	.082
	Present	11	55	17	85	

Anger problems	Absent	14	70	4	20	.004**
	Present	6	30	16	80	

Concentration problems	Absent	8	40	3	15	.155
	Present	12	60	17	85	

Heightened awareness	Absent	6	30	3	15	.451
	Present	14	70	17	85	

Startle reaction	Absent	1	5	2	10	1.00
	Present	19	95	18	90	

At least two hyper-arousal symptoms	Absent	2	10	0	0	.487
	Present	18	90	20	100	

Daily transport difficulties	Absent	2	10	4	20	.661
	Present	18	90	16	80	

a *n* = 20 for each group.

b TSQ: Trauma Screener Questionnaire (Brewin et al., 2002).

* *p* < .05.

** *p* < .01.
